# Functional and structural deficits at accumbens synapses in a mouse model of Fragile X

**DOI:** 10.3389/fncel.2015.00100

**Published:** 2015-03-26

**Authors:** Daniela Neuhofer, Christopher M. Henstridge, Barna Dudok, Marja Sepers, Olivier Lassalle, István Katona, Olivier J. Manzoni

**Affiliations:** ^1^INSERM U901Marseille, France; ^2^INMEDMarseille, France; ^3^Université de Aix-Marseille, UMR S901Marseille, France; ^4^Momentum Laboratory of Neurobiology, Institute of Experimental Medicine, Hungarian Academy of SciencesBudapest, Hungary; ^5^School of Ph.D. Studies, Semmelweis UniversityBudapest, Hungary; ^6^Department of Psychiatry, University of British ColumbiaVancouver, Canada

**Keywords:** synaptic plasticity, spike timing-dependent plasticity, accumbens, Fragile X, dendritic spines, autism

## Abstract

Fragile X is the most common cause of inherited intellectual disability and a leading cause of autism. The disease is caused by mutation of a single X-linked gene called *fmr1* that codes for the Fragile X mental retardation protein (FMRP), a 71 kDa protein, which acts mainly as a translation inhibitor. Fragile X patients suffer from cognitive and emotional deficits that coincide with abnormalities in dendritic spines. Changes in spine morphology are often associated with altered excitatory transmission and long-term plasticity, the most prominent deficit in *fmr1-/y* mice. The nucleus accumbens, a central part of the mesocortico-limbic reward pathway, is now considered as a core structure in the control of social behaviors. Although the socio-affective impairments observed in Fragile X suggest dysfunctions in the accumbens, the impact of the lack of FMRP on accumbal synapses has scarcely been studied. Here we report for the first time a new spike timing-dependent plasticity paradigm that reliably triggers NMDAR-dependent long-term potentiation (LTP) of excitatory afferent inputs of medium spiny neurons (MSN) in the nucleus accumbens core region. Notably, we discovered that this LTP was completely absent in *fmr1-/y* mice. In the *fmr1-/y* accumbens intrinsic membrane properties of MSNs and basal excitatory neurotransmission remained intact in the *fmr1-/y* accumbens but the deficit in LTP was accompanied by an increase in evoked AMPA/NMDA ratio and a concomitant reduction of spontaneous NMDAR-mediated currents. In agreement with these physiological findings, we found significantly more filopodial spines in *fmr1-/y* mice by using an ultrastructural electron microscopic analysis of accumbens core medium spiny neuron spines. Surprisingly, spine elongation was specifically due to the longer longitudinal axis and larger area of spine necks, whereas spine head morphology and postsynaptic density size on spine heads remained unaffected in the *fmr1-/y* accumbens. These findings together reveal new structural and functional synaptic deficits in Fragile X.

## Introduction

Fragile X is the most common monogenetic cause of inherited intellectual disability and a leading cause of autism. The disease is caused by mutation of a single X-linked gene called *fmr1* (Verkerk et al., 1991). The Fragile X mental retardation protein (FMRP) is a 71 kDa protein which regulates the transport and translation of more than 850 mRNAs in the brain and especially in synapses (Ronesi and Huber, 2008; Darnell et al., 2011; Maurin et al., 2014). Fragile X patients suffer from intellectual disability and neuropsychiatric problems such as social anxiety, attention-deficit hyperactivity and sensory hypersensitivity (de Vries et al., 1998; Tranfaglia, 2011). The *fmr1-/y* mice display behavioral phenotypes that correspond to many of the symptoms found in FRAX patients (Kooy, 2003). One key pathological feature of the disease is the presence of distinctive spine abnormalities, which have been found in the post-mortem tissue of Fragile X patients as well as in *fmr1-/y* mice (Comery et al., 1997; Irwin et al., 2000, 2001). This morphological abnormality coincides with altered synaptic plasticity, which was first described at hippocampal excitatory synapses, in the form of exaggerated protein translation- and mGluR-dependent long-term depression (mGluR-LTD; Bear et al., 2004). Since then many different forms of brain region-specific and age-dependent deficits in synaptic plasticity have been described (for reviews see Martin and Huntsman, 2012; Sidorov et al., 2013).

The nucleus accumbens, the ventral part of the striatum, has been extensively studied in the context of reward-related behaviors (Gipson et al., 2014). Its role in rewarding social behaviors and social interactions has recently been highlighted (Wallace et al., 2009; Dölen et al., 2013; Gunaydin et al., 2014). Although altered social behavior and interactions are core symptoms in Fragile X patients, how morphological and neurophysiological maladaptation of accumbal synapses participate in the disease remains poorly understood (Jung et al., 2012). This is critically important, however, in light of the key physiological regulatory function of the excitatory afferent pathways and their synaptic integration and persistent modifications in the control of social reward-related and goal-directed behaviors (McGinty and Grace, 2008; Sesack and Grace, 2010; Grueter et al., 2012; Papp et al., 2012). The ultrastructural changes accompanying synaptic plasticity deficits in this brain region in the mouse model of Fragile X syndrome remain also obscure. Thus far, only one study suggested impaired dendrites and spines in the accumbens of *fmr1-/y* mice (Jung et al., 2012). A detailed ultrastructural analysis of the morphological parameters of dendritic spines and their afferent excitatory synapses on spine heads are still lacking in the *fmr1-/y* mice.

In this study, we aimed to identify previously undisclosed alterations in long-term potentiation (LTP) and spine architecture at accumbens excitatory synapses in *fmr1-/y* mice. We discovered an impaired spike-timing-dependent LTP in medium spiny neurons located in the accumbens core region of *fmr1-/y* mice, which was associated with a higher ratio of evoked synaptic AMPAR- and NMDAR-mediated currents. In accordance with the idea that functional deficits occur together with structural alterations of the synapse, an ultrastructural analysis by electron microscopy revealed marked alterations in postsynaptic spine number and structure. Most importantly, long torturous spines were much more common in the accumbens core region of *fmr1-/y* mice, which was the result of a specific elongation of the spine neck, but not the spine head. Together these data shed new light on the functional and structural alterations in the accumbens of *fmr1-/y* mice and suggest new synaptic substrates for some of the behavioral deficits observed in Fragile X.

## Materials and Methods

### Animals

Animals were treated in compliance with the European Communities Council Directive (86/609/EEC) and the United States National Institutes of Health Guide for the Care and Use of Laboratory Animals. All animals were housed, grouped and acclimated to laboratory conditions for 4 days before experiments with 12 h light/dark cycles and access to food and water *ad libitum*.

### Slice Preparation and Electrophysiology

#### Slice Preparation

Adult male *fmr1-/y* mice on a C57Bl6/J genetic background aged between 60 and 180 postnatal days were used, with wild-type littermates used as control group (Jung et al., 2012). They were anesthetized with isoflurane and decapitated according to institutional regulations. The brain was sliced (300 μm) in the coronal plane with a vibratome (Integraslice, Campden Instruments, Loughborough, UK) in a sucrose-based solution at 4°C (in mM: 87 NaCl, 75 sucrose, 25 glucose, 2.5 KCl, 4 MgCl_2_, 0.5 CaCl_2,_ 23 NaHCO_3_ and 1.25 NaH_2_PO_4_). Immediately after cutting, slices were stored for 1 h at 32°C in a low calcium artificial cerebrospinal fluid (low Ca^2+^ACSF) that contained (in mM): 130 NaCl, 11 Glucose, 2.5 KCl, 2.4 MgCl_2_, 1.2 CaCl_2_, 23 NaHCO_3_, 1.2 NaH_2_PO_4_, and was equilibrated with 95% O_2_/5% CO_2_ and then at room temperature until the time of recording.

#### Electrophysiology

Whole cell patch-clamp of visualized MSN and field potential recordings were made in coronal slices containing the ventral striatum as previously described (Robbe et al., 2002c). Recordings were made in the medial ventral accumbens core close to the anterior commissure (Robbe et al., 2002c).

For recording, slices were placed in the recording chamber and superfused (1.5–2 ml/min) with ACSF (same as low Ca^2+^ ACSF with the following exception: 2.4 mM CaCl_2_ and 1.2 mM MgCl_2_). All experiments were done at 32°C. The superfusion medium contained picrotoxin (100 μM) to block gamma-aminobutyric acid types A (GABA-A) receptors. All drugs were added at the final concentration to the superfusion medium. For whole cell patch-clamp experiments, neurons were visualized using an upright microscope with infrared illumination. The intracellular solution was based on K^+^ gluconate (in mM: 145 K^+^ gluconate, 3 NaCl, 1 MgCl_2_, 1 EGTA, 0.3 CaCl_2_, 2 Na_2_^+^ ATP, and 0.3 Na^+^ GTP, 0.2 cAMP, buffered with 10 HEPES. To quantify the AMPA/NMDA ratio we used a CH_3_O_3_SCs-based solution (in mM:128 CH_3_O_3_SCs, 20 NaCl, 1 MgCl_2_, 1 EGTA, 0.3 CaCl_2_, 2 Na_2_^+^ATP, and 0.3 Na^+^ GTP, 0.2 cAMP, buffered with 10 HEPES, pH 7.2, osmolarity 290–300 mOsm. The pH was adjusted to 7.2 and osmolarity to 290–300 mOsm. Electrode resistance was 4–6 MOhms.

A −2 mV hyperpolarizing pulse was applied before each evoked EPSC in order to evaluate the access resistance and those experiments in which this parameter changed >25% were rejected. Access resistance compensation was not used and acceptable access resistance was <30 MOhms. The potential reference of the amplifier was adjusted to zero prior to breaking into the cell. Cells were held at −76 mV. Current-voltage (I-V) curves were made by a series of hyperpolarizing to depolarizing current steps immediately after breaking into the cell. Membrane resistance was estimated from the I–V curve around resting membrane potential (Kasanetz and Manzoni, 2009).

Whole cell patch-clamp recordings were performed with an Axopatch-200B amplifier. Data were low pass filtered at 2 kHz, digitized (10 kHz, DigiData 1440A, Axon Instrument), collected using Clampex 10.2 and analyzed using Clampfit 10.2 (all from Molecular Device, Sunnyvale, USA). Both fEPSP area and amplitude were analyzed. Stimulation was performed with a glass electrode filled with ACSF and placed ~200 μm in the dorsal-medial direction of the recorded cell. The stimulus intensity was adjusted around 60% of maximal intensity after performing an input-output curve (baseline EPSC amplitudes ranged between 50–150 pA). Stimulation frequency was set at 0.1 Hz.

STDP induction: The STDP induction protocol was performed in the current clamp configuration. The pre-post STDP protocol consisted of the baseline electrical stimulation followed by a supra-threshold depolarization of the recorded neuron to elicit an action potential. The time of the depolarization was adjusted for each cell to achieve a delay of 25 ms between the beginning of the EPSP and the action potential. This protocol was then delivered 60 times at 0.1 Hz.

E-S Coupling: For ES-Coupling analysis, ten traces were recorded for each stimulus. The value of ES-Coupling obtained for each animal was calculated by averaging the spiking probability corresponding to each class of EPSP slope. EPSP slopes were measured during the first 2 ms, sorted in 0.5 mV/ms bins, and the firing probability was determined for each bin.

Spontaneous EPSCs (sEPSCs) were recorded at −76 mV (AMPAR-mediated sEPCS) or +40 mV (NMDAR-mediated sEPSC) in whole cell voltage-clamp configuration using Axoscope 10 (Molecular Devices). sEPSCs were filtered at 2 kHz and digitized at 20 kHz. sEPSCs amplitude and inter-interval time were analyzed with Axograph X using a double exponential template: f(t) = exp(−t/rise) + exp(−t/decay), rise = 0.5 ms or 3 ms and decay = 3 ms or 10 ms, for AMPAR- and NMDAR-mediated EPSCs, respectively. The threshold of amplitude detection was set at 5 pA or 2 pA for AMPAR- and NMDAR-mediated EPSCs, respectively.

### Data Acquisition and Analysis

The magnitude of plasticity was calculated 20–30 min after the potentiation protocol as percentage of baseline responses. To determine the AMPA/NMDA ratio, the cells were voltage clamped to +40 mV and a stable dual response (AMPA + NMDA current) to afferent stimulation was recorded. The AMPAR EPSC was isolated after bath application of the NMDAR antagonist D-2-amino- 5-phosphonovaleric acid (D-APV, 50 μM). The NMDAR EPSC was obtained by digital subtraction of the AMPAR EPSC from the dual response. Spontaneous EPSCs were analyzed with Axograph X (Axograph). Statistical analysis of data was performed with GraphPad Prism (GraphPad Software Inc., La Jolla, CA) using tests indicated in the main text after outlier subtraction. All values are given as mean ± standard error and statistical significance was set at *p < 0.05 and **p < 0.01.

### Anatomy

#### Animals, Perfusion and Preparation of Tissue Sections

All animal experiments were approved by the Hungarian Committee of the Scientific Ethics of Animal Research (license number: XIV-1-001/2332-4/2012), and were carried out according to the Hungarian Act of Animal Care and Experimentation (1998, XXVIII, Section 243/1998), which are in accordance with the European Communities Council Directive of 24 November 1986 (86/609/EEC; Section 243/1998). All efforts were made to minimize pain and suffering and to reduce the number of animals used. Adult male C57BL/6 J mice [three wild-type and three *fmr1-/y* mice (8 weeks old) were deeply anesthetized with a mixture of ketamine–xylazine (25 mg/ml ketamine, 5 mg/ml xylazine, 0.1% w/w pipolphen in H_2_O; 1 ml/100 g, i.p.). Animals were then perfused transcardially with 0.9% saline for 2 min, followed by 100 ml of fixative containing 4% paraformaldehyde and 0.1% glutaraldehyde in 0.1 M phosphate buffer (PB), pH 7.4, for 20 min. After perfusion, the brain was removed from the skull, cut into blocks, post-fixed for 2 h and washed in PB. The blocks containing the ventral striatum were sliced into 50-μm-thick coronal sections of the brain with a Leica VTS-1000 vibratome (Vibratome, St. Louis, MO).

#### Immunogold Labeling

Immunostaining does not feature in this report, however the tissue analyzed underwent immunogold labeling, which is fully detailed in our previous study (Jung et al., 2012). Briefly, after slicing and extensive washing in 0.1 M PB, the sections were incubated in 10% sucrose for 15 min and 30% sucrose overnight, followed by freeze thawing over liquid nitrogen four times. Subsequently, all washing steps and dilutions of the antibodies were performed in 0.05 M tris-buffered saline (TBS), pH 7.4. After extensive washing in TBS, the sections were blocked in 5% normal goat serum for 45 min and then incubated with an antibody against diacylglycerol lipase-α for a minimum of 48 h at 4°C. The sections were washed extensively in TBS before incubation in 0.8 nm gold-conjugated goat anti-rabbit secondary antibody (1:50; AURION, Wageningen, The Netherlands), overnight at 4°C. Then sections were silver intensified using the silver enhancement system R-GENT SE-EM according to the kit protocol (AURION). After development, the sections were treated with osmium tetroxide (0.5%) in PB for 20 min at 4°C and dehydrated in an ascending series of ethanol and acetonitrile, before being embedded in Durcupan (ACM, Fluka, Buchs, Switzerland). During dehydration, sections were treated with 1% uranyl acetate in 70% ethanol for 15 min at 4°C. For electron microscopy analysis, areas of interest in the ventral striatum core were removed from Durcupan embedded sections, then re-embedded and re-sectioned. Ultrathin (60 nm) sections were collected on Formvar-coated single-slot grids and stained with lead citrate. Electron micrographs were taken at 20,000 or 40,000× magnifications with a Hitachi 7100 electron microscope (Tokyo, Japan). An experimenter blind to the genotype of the mice performed image collection and data analysis.

#### Synapse Density Analysis

To assess the density of excitatory synapses in the neuropil of the accumbens, 50 electron micrographs were captured randomly at 20,000× magnification for each animal by moving two fields of view in the *x*-direction and one field of view in the *y*-direction between images. Clearly identifiable postsynaptic densities (PSDs) were used as an initial identification of putative synapses. Synapses were only counted when standard morphological parameters were met, including clear pre- and post-synaptic compartments and a distinct synaptic cleft. The number of synapses in each image was then divided by the area of each image to generate a density value of PSDs/micron squared (PSD/μm^2^). To compare the densities between genotype, a mean density value was generated for each animal from the 50 images and an unpaired Student’s *t*-test performed with the 3 wild-type vs. 3 *fmr1-/y* mean densities. Density was also assessed using the 3D dissector approach, as described by Geinisman et al. (1996). Serial, ultrathin 60 nm sections were collected from all six animals and three images series captured at random at 20,000× magnification. A dissector frame with an area of 21.89 μm^2^ was applied to all images and only synapses within the frame or dissected by the inclusion lines of the frame were counted. The volume of each serial image stack was 1.13 μm^3^ and consisted of 6 serial sections. Each image was used initially as the reference image and then the “look up” image and synapses were only counted if found in the “look up” image and not in the reference image. Dividing all the synapses from each animal across the three stacks by the total dissector volume and expressed as PSDs/μm^3^ generated synaptic density values. To compare the densities between genotype, a mean density value was generated for each animal and an unpaired Student’s *t*-test performed with the 3 wild-type vs. 3 *fmr1-/y* mean densities.

#### PSD Size Analysis

Length of PSD was measured in 100 randomly chosen synapses per animal, captured at 40,000× magnification. To ensure unbiased sampling, on average the section was imaged following two fields of view movement in the *x*-direction and one field of view in the *y*-direction between images, regardless of the shape and size of the synapse. However, synapses were only imaged when standard morphological parameters were met, including clear pre- and post-synaptic compartments and a distinct synaptic cleft. Mean lengths were generated per animal and an unpaired Student’s *t*-test performed with the 3 wild-type vs. 3 *fmr1-/y* mean lengths. To ensure there were no subtle differences in the spread of PSD lengths between genotypes, the data was pooled within genotypes and then binned according to size. To assess any difference in the spread of the data, a chi-squared test was run. PSD area was also calculated using a 3D approach as described above in stacks of images 360 nm deep and all PSDs within found completely enclosed within this depth were analyzed (*n* = 4–9 PSDs per animal). Assuming that PSD exists as a disc, the length of the dissected PSD was measured in each serial section and multiplied by the section depth (60 nm) to give the PSD area for each serial image. Total PSD area (μm^2^) was generated by adding these dissected areas together. To compare the PSD area between genotype, a mean density value was generated for each animal and an unpaired Student’s *t*-test performed with the 3 wild-type vs. 3 *fmr1-/y* mean values.

#### Spine and Bouton Morphological Analysis

To assess spine morphology, approximately 75 intact spines with a clear dendritic base and excitatory synapse were imaged at 40,000× magnification, per animal. Spine length was measured in nanometers (nm) from the base of the neck to the tip of the head using the line measurement tool in ImageJ. Assuming spines to exist as a “ball and stick” representing the head and neck respectively, the spine head boundary was estimated by the continued convex shape of the head. Spine neck length was measured from the base of the spine to the base of the head boundary. Spine neck diameter was measured at the thinnest appearing point along the neck. Head length was measured from the base of the head boundary to the tip of the head. Spine area was calculated using ImageJ by outlining the entire spine boundary and drawing a straight line across the base of the neck. Head and neck area were calculated by outlining the neck and head boundaries using ImageJ.

To assess presynaptic alterations, 50 excitatory axospinous synapses were imaged at random, per animal. Active zone length was measured along the contour of the presynaptic terminal membrane and was determined by the weakly electron dense active zone directly appositional to the postsynaptic PSD. Total vesicle number was calculated by counting all intact vesicles in the presynaptic terminal.

For each parameter, all values from each animal were used to create a mean value per animal. The 3 wild-type values were compared to the 3 *fmr1-/y* values using an unpaired Student’s *t*-test. When mean values of individual animals belonging to the same genotype were similar the distribution of the values was compared by pooling all values per genotype and binning the data according to size. Chi-squared tests were used to analyze differences in the data spread between genotypes. To assess correlation between morphological parameters, all raw data points were used to generate a scatterplot and Pearson’s correlation *R*^2^ values were generated for each association to characterize the significance of correlation, when *p* < 0.05.

All measurements were performed using ImageJ measurement tools, figures prepared in PhotoShop and statistical tests (unpaired Student’s *t*-test, Pearson’s correlation, Kolmogorov-Smirnov and chi-squared test) were performed using GraphPad Prism 4.

## Results

### NMDAR-Dependent Spike-Timing-Dependent Potentiation in Afferent Synapses of Medium Spiny Neurons in the Nucleus Acccumbens Core Region

**Figure 1:**
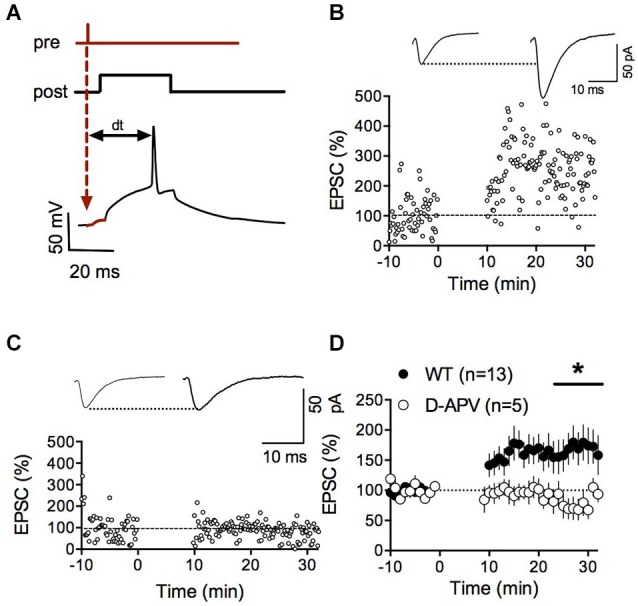
**NMDAR-dependent spike timing-dependent potentiation (LTP) in accumbens medium spiny neurons. (A)** Schematic representation of the pre-post protocol used to induce LTP. After the presynaptic stimulation the postsynaptic cell was depolarized for 30 ms to elicit an action potential. The time delay between presynaptic stimulation and the elicited spike was set to 25 ms. **(B)** Representative experiment illustrating the induction of LTP. Inset shows EPSCS sampled during the 10 min baseline and 20 min after LTP induction respectively. **(C)** Representative experiment showing that LTP in WT mice was abolished by the application of the NMDAR-antagonist D-APV (50 μM). **(D)** Summary of LTP experiments with (white circles) and without D-APV (black circles). LTP was blocked by application of 50 μM D-APV (*p* = 0.0441 Mann-Whitney test).

Numerous forms of activity-dependent LTD are expressed by accumbens synapses (Robbe et al., 2002a,b,c; Grueter et al., 2010). Reports of LTP are less common (Pennartz et al., 1993; Kombian and Malenka, 1994; Schramm et al., 2002; Schotanus and Chergui, 2008a) and complicated by the poor reliability of the induction protocols (Robbe et al., 2002b; Ji and Martin, 2012). Spike-timing-dependent plasticity (STDP) is widely considered as a physiologically relevant paradigm to trigger synaptic plasticity at central synapses (Dan and Poo, 2004; Caporale and Dan, 2008). While STDP has been well described for excitatory synapses in the dorsal striatum (Shen et al., 2008; Fino and Venance, 2010; Paille et al., 2013), a reliable LTP inducing STDP protocol for the ventral striatum is still lacking (Ji and Martin, 2012). Therefore, we first systematically searched for a consistent STDP protocol for accumbens medium spiny neurons (MSN) in adult wild-type mice based on induction parameters published *a priori* (Fino et al., 2005). When presynaptic stimulation was followed by a 30 ms postsynaptic depolarization eliciting a spike (dt = 25 ms), we observed a strong potentiation of synaptic efficacy (*p* = 0.0134 Wilcoxon matched pairs signed rank test, Figures 1A,B). Typically, LTP depends on the activation of postsynaptic NMDAR (Markram et al., 1997; Dan and Poo, 2004; Nevian and Sakmann, 2006). Accordingly, we found that bath application of the specific NMDAR antagonist D-APV completely prevented LTP (*p* = 0.0441, Mann-Whitney test; Figures 1C,D). Together these experiments demonstrate that accumbens excitatory synapses can reliably express NMDAR-dependent LTP with induction parameters specific for this synapse type.

### Adult *fmr1-/y* Accumbens Neurons Lack LTP and Show Augmented AMPA/NMDA Ratios

Previous work from our laboratory has shown that LTD mediated by the mGluR5/endocannabinoid-signaling complex is absent in *fmr1-/y* mice (Jung et al., 2012). Reports of altered LTP in cortical areas abound (Padmashri et al., 2013; Sidorov et al., 2013; Boda et al., 2014; Chen et al., 2014; Franklin et al., 2014; Yang et al., 2014). In particular, a previous study by Meredith and collaborators revealed a strong impairment of STDP in the prefrontal cortex superficial pyramidal cells in *fmr1-/y* mice (Meredith et al., 2007). Various impairments of LTP and LTD in the accumbens could participate in the social deficits observed in *fmr1-/y* mice and Fragile X patients (Oddi et al., 2013).

**Figure 2:**
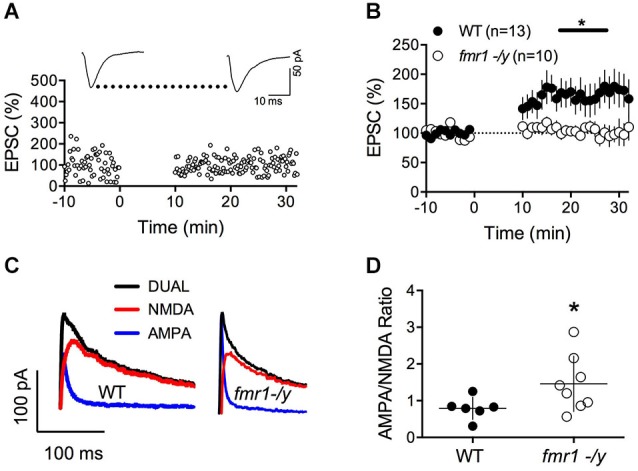
**Lack of LTP and augmented AMPA/NMDA ratio in accumbens MSN of *fmr1-/y mice*. (A)** Representative experiment illustrating the lack of LTP in *fmr1-/y* mice. Inset shows EPSCs averaged over 10 min baseline and 20 min after the induction protocol respectively. **(B)** Averaged time-courses of LTP experiments for both genotypes. LTP was absent in *fmr1-/y* mice (*p* = 0.0415 Mann-Whitney test) **(C)** Representative current traces of a wild type (left) and *fmr1-/y* (right) MSN voltage clamped at −40 mV to illustrate the computation of A/N ratios. Black: Dual AMPA and NMDA response. Blue: isolated AMPA response after application of d-APV (50 μM). Red: NMDA response extracted via subtraction of AMPA response from the dual response. **(D)** A/N ratios were larger in *fmr1-/y* mice (*p* = 0.043, Mann-Whitney test).

To directly address this possibility and extend our previous work on LTD, we tested whether we could evoke LTP in *fmr1-/y* mice. Using our new STDP protocol, we found that NMDAR-mediated LTP was ablated in accumbens MSN of *fmr1-/y* mice compared to their wild type littermates (*p* = 0.0415 Mann-Whitney test; Figures 2A,B). In physiological and pathological conditions, long-term plasticity and the ratio of evoked synaptic AMPA/NMDA ratio often covariate (Gocel and Larson, 2012; Gipson et al., 2014). Ample evidence points toward protracted changes in the AMPA/NMDA ratio in rodent models of mental disability and autism. For example, in the *in utero* valproate exposure model of autism, we recently reported that adult rats had impaired prefrontal LTP and enhanced AMPA/NMDA ratio (Martin and Manzoni, 2014). Therefore, we next quantified and compared the ratio of evoked synaptic AMPAR and NMDAR currents (AMPA/NMDA ratio). We found that this index was augmented in *fmr1-/y* mice compared to their wild-type littermates (*p* = 0.043 Mann-Whitney test; Figures 2C,D).

### Intrinsic Properties and Synaptic Parameters of Accumbens Medium Spiny Neurons of Adult *fmr1-/y* and Wild-Type Mice

**Figure 3:**
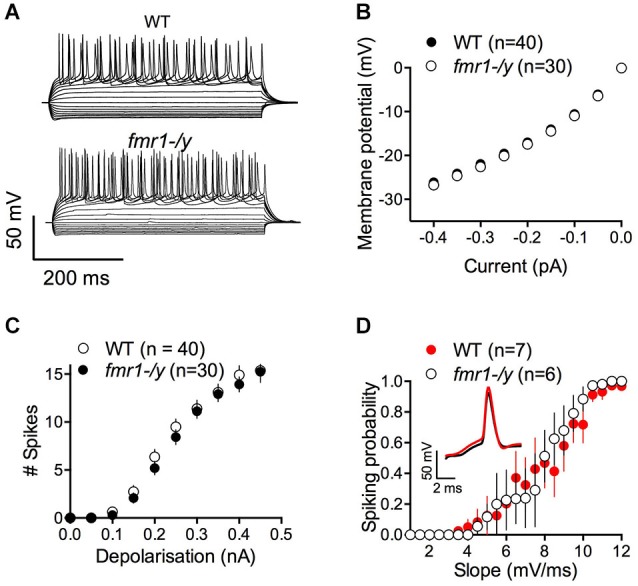
**Similar excitability profile in wild-type and *fmr1-/y medium spiny neurons*. (A)** Representative traces of voltage responses to somatic current injections of a wild-type (upper panel, WT) and *fmr1-/y* (lower panel) medium spiny neuron. **(B)** The voltage responses to hyperpolarizing current pulses revealed no differences in input resistance or inward rectification between the two genotypes (*p* = 0.2770, two way ANOVA; WT *n* = 40, black symbols; *fmr1-/y n* = 30, white symbols). **(C)** The number of action potentials as a function of depolarizing current injections was similar (*p* = 0.1272, two way ANOVA). **(D)** The firing probability plotted as a function of the EPSP slope revealed no changes in the Excitation-Spike coupling (*p* = 0.1488, two way ANOVA; WT *n* = 8, black symbols; *fmr1-/y n* = 5, white symbols).

The lack of LTP in *fmr1-/y* mice could be caused by alterations of intrinsic and/or firing properties of the MSNs. Thus, we compared some of the basic properties of these neurons. Independently of their genotypes, all recorded MSNs showed similar membrane response profiles in response to a series of somatic current steps as shown in superimposable I-V plots (*p* = 0.2770, two way ANOVA; Figures 3A,B). The number of action potentials in response to somatic current steps was also similar in wild-type and *fmr1-/y* mice (*p* = 0.1272, two way ANOVA; Figure 3C). Furthermore the lack of LTP cannot be explained by different spiking in response to the LTP protocol. The jitter i.e., the standard deviation of spike timing was 1.25 ± 0.38 SD and 1.308 ± 0.93 SD for wild-type and in *fmr1-/y* mice respectively (*p* = 0.8503, Student’s unpaired *t*-test). We also determined the excitatory postsynaptic potential-spike coupling (or E-S coupling) to directly evaluate how synaptic excitation is integrated to generate an action potential in wild-type and *fmr1-/y* littermates (Thomazeau et al., 2014). We found that the E-S coupling was similar in wild-type and in *fmr1-/y* mice (*p* = 0.1488, two way ANOVA, Figure 3D). We conclude that the lack of FMRP in *fmr1-/y* mice has no major effect on excitatory synaptic integration in accumbens MSNs. More generally, the data indicate that the lack of FMRP expression did not affect on the intrinsic properties of accumbens MSNs.

**Figure 4:**
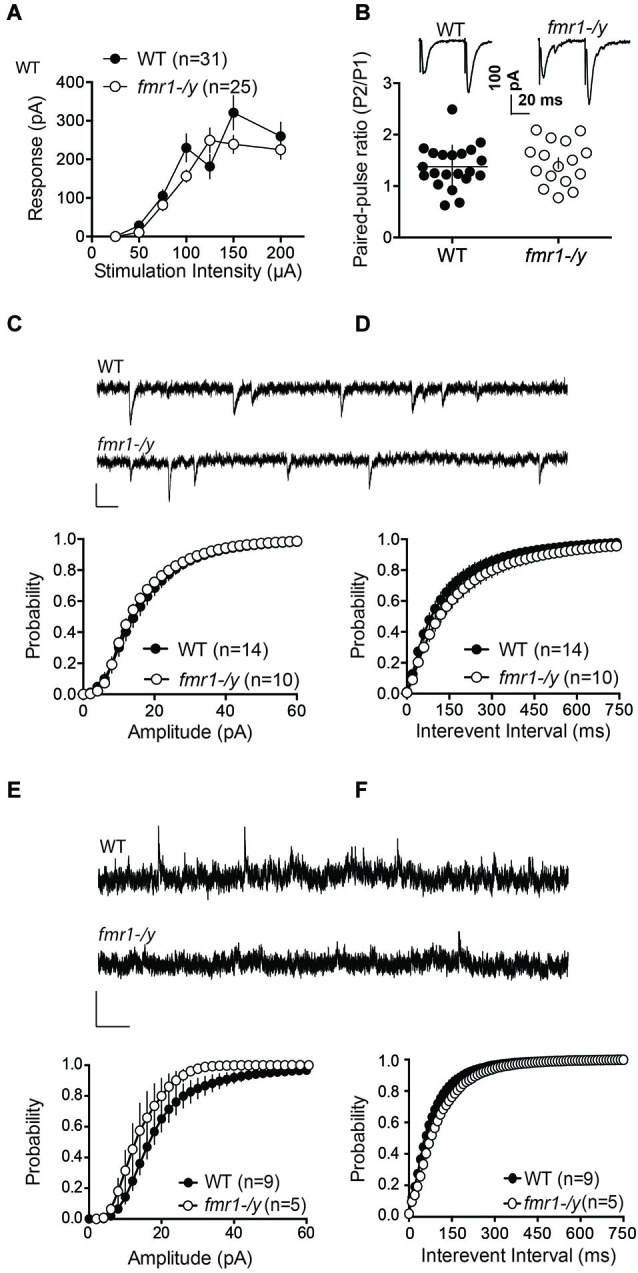
**Glutamatergic transmission parameters in the nucleus accumbens of wild-type and *fmr-/y* mice. (A)** Average field responses to electric stimulation of increasing intensity did not reveal a significant difference in synaptic excitability between the two genotypes. The overall excitability was not significantly different between the two genotypes (*p* = 0.1436, 2-way ANOVA, the number of animals tested differed between stimulation intensities *n* = 14–31, data not shown). **(B)** Example traces illustration the response to paired stimulations for a wild type (upper trace) and a *fmr1-/y* mouse (lower trace). The comparison of the median paired-pulse ratios for a stimulus interval of 50ms revealed no significant difference between the two genotypes (*p* = 0.4354, WT *n* = 21, *fmr1-/y n* = 16, students *t*-test). **(C)** Sample traces from accumbens MSN clamped at −70 mV from wild type and *fmr1/y* animals (scale bar: 50 ms, 20 pA). The cumulative probability distribution of AMPAR sEPSCs amplitudes revealed no differences between the two genotypes (Kolmogorov-Smirnov-test; WT *n* = 14, black symbols; *fmr1-/y n* = 10, white symbols). **(D)** The cumulative probability distribution of AMPAR sEPSCs inter-event-intervals revealed no differences in spontaneous synaptic transmission between the two genotypes (Kolmogorov-Smirnov-test; WT *n* = 14, black circles; *fmr1-/y n* = 10, white circles). **(E)** Sample traces from accumbens MSN clamped at +40 mV from wild type and *fmr1-/y* animals (scale bar: 2 s, 50 pA). The cumulative probability distribution of NMDAR sEPSCs amplitudes revealed a significant difference between the two genotypes (Kolmogorov-Smirnov-test *p* < 0.0001); WT *n* = 9, black symbols; *fmr1-/y n* = 5 white symbols). **(F)** The cumulative probability distribution of NMDAR sEPSCs inter-event-intervals revealed no differences in spontaneous synaptic transmission between the two genotypes (Kolmogorov-Smirnov-test; WT *n* = 9, black circles; *fmr1-/y n* = 5, white circles).

We next measured field EPSPs (fEPSP) of accumbens MSNs to build input-output profiles in the two genotypes. fEPSPs evoked by electrical stimulation showed a consistent profile distribution in response to increasing stimulation intensity across different slices and mice (Figure 4A). Furthermore, input-output curves from wild-type and *fmr1-/y* littermates were identical. The data show that the excitability of accumbens MSN synapses was unaltered (Figure 4A). Additionally, the paired pulse ratio, a form of short-term synaptic plasticity that depends on release probability of glutamate, was identical in both genotypes (Figure 4B). These data suggest that the lack of LTP is unlikely due to a reduction of the number of synapses recruited during the induction of synaptic plasticity in *fmr1-/y* accumbens synapses.

Our present observation of augmented AMPA/NMDA ratio (Figures 2C,D) can be explained by synaptic insertion of additional AMPAR or/and enhanced AMPAR conductance in *fmr1-/y* mice or/and reduction of NMDAR conductance. To test for this possibility, we compared quantal events by recording spontaneous AMPAR- and NMDAR-mediated EPSCs (sEPSC) in accumbens MSNs from both wild-type and *fmr1-/y* littermate neurons. Figures 4C,D shows the summary cumulative distribution of the amplitude in the two groups (Figure 4C). Both the distribution and the mean amplitude of spontaneous events were similar in the two genotypes. At resting membrane potential (−70 mV), these events are principally mediated by AMPAR therefore the lack of FMRP does not appear to affect AMPAR currents in accumbens MSN. We next compared the frequency of sEPSC by comparing the cumulative distribution of the interval between events (Figure 4D). Both genotypes had a similar distribution and average inter-event intervals.

Figure 4E shows the summary cumulative distribution of NMDAR-sEPSC amplitude in the two genotypes. There was a shift to the left of the distribution of the amplitude of spontaneous NMDAR-mediated events in *fmr1-/y* mice compared to wild-type littermates. We compared the frequency of sEPSC by comparing the cumulative distribution of the interval between events (Figure 4F). Both genotypes had a similar distribution and average interval between NMDAR-mediated events. These data are compatible with a reduction in postsynaptic NMDAR density and/or conductance in *fmr1-/y* mice.

These data together with normal intrinsic properties (Figure 3) and unchanged input/output curves (Figure 4A) suggest that the profound impairment of LTP observed in *fmr1-/y* mice could be linked to a modification of synaptic NMDAR content.

### Altered Dendritic Spines of Medium Spiny Neurons in the Core Region of Nucleus Accumbens of Adult *fmr1-/y* Mice

**Figure 5:**
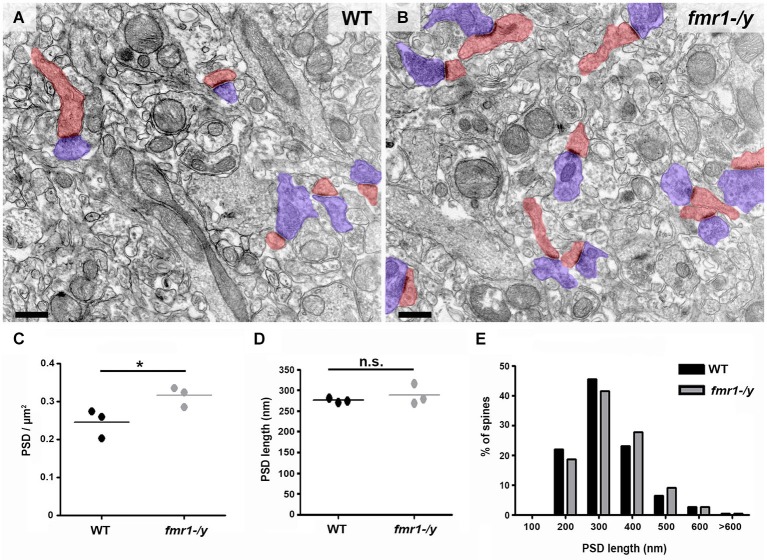
**Higher density of excitatory synapses in the accumbens of *fmr1-/y* mice**. Electron micrographs captured in the core of the nucleus accumbens of wild-type **(A)** and *fmr1-/y* mice **(B)** reveal the scattered distribution and different density of excitatory synapses in the two genotypes. These putative glutamatergic synapses were identified by the clear presence of a PSD as well as pre- (blue) and postsynaptic (red) compartments. Scale bars = 500 nm. **(C)** Mean PSD density values from 50 images per animal revealed a significant increase in excitatory synapse density in *fmr1-/y* mice (*n* = 3) compared to wild-type (*n* = 3). Unpaired *t*-test, *p* = 0.049. **(D)** Mean PSD lengths measured from 100 synapses per animal (*n* = 3) revealed identical PSD length in the two genotypes. Unpaired *t*-test, *p* = 0.422. **(E)** Separation of PSD length values into 100 nm bins followed by a Chi-squared test, revealed no difference in PSD length distribution, *p* = 0.670.

Recent results have demonstrated a tight correlation between spine morphology and synaptic strength (Araya et al., 2014; Tønnesen et al., 2014). Therefore, we next searched for structural alterations that could contribute to the impaired synaptic plasticity in *fmr1-/y*. Dendritic spine anomalies are common in neuropsychiatric diseases and constitute a core feature of intellectual disability (Penzes et al., 2011). A common finding in both human patients and mouse models of FRAX, is the higher number of spines in multiple brain regions (He and Portera-Cailliau, 2013). In line with these findings, we found that the density of excitatory synapses innervating spine heads was significantly increased on average by 28% in the accumbens of *fmr1-/y* mice (Figures 5A–C). Postsynaptic density (PSD) distribution in *fmr1-/y* mice (0.32 ± 0.02 PSD/μm^2^) was significantly denser than wild-type accumbens (0.25 ± 0.02 PSD/μm^2^; *p* = 0.049). In contrast, PSD length was similar (wild-type = 275 ± 3 nm, *fmr1-/y* = 289 ± 15 nm; *p* = 0.42) between genotypes (*n* = 300 synapses per genotype), suggesting that the increase in synapse number in the absence of FMRP is not the consequence of a potential sampling error of differentially sized PSDs (Figure 5D,E). To corroborate these observations, we also performed a 3D stereological approach to more accurately assess PSD density and size. This experiment confirmed our 2D analysis, showing an increased PSD density in the *fmr1-/y* mice (wild-type = 1.1 ± 0.2 PSDs/μm^2^, *fmr1-/y* = 1.7 ± 0.04 PSDs/μm^2^; *p* = 0.046), yet confirmed the similarity in the PSD area (wild-type = 0.036 ± 0.004 μm^2^, *fmr1-/y* = 0.039 ± 0.002 μm^2^; *p* = 0.64).

**Figure 6:**
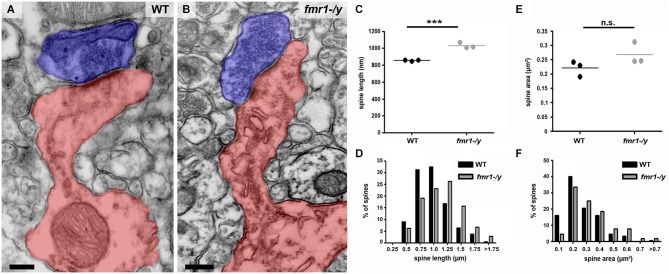
**Higher incidence of elongated spines in the accumbens of *fmr1-/y* mice. (A)** Electron micrograph of a typical “mushroom” spine from the accumbens of a wild-type mouse. Scale bar = 100 nm. **(B)** Electron micrograph of longer, spines found in the accumbens of an *fmr1-/y* mouse. Presynaptic terminals are highlighted in blue, postsynaptic spine(s) labeled in red. Scale bar = 100 nm. **(C)** Analysis of mean spine length (*n* = 3 WT, *n* = 3 *fmr1-/y*) uncovered the presence of longer spines in the *fmr1-/y* mice. Unpaired *t*-test, *p* = 0.001. **(D)** Binned data from all 221 wild-type and 224 *fmr1-/y* spines reveals an increase in the number of long spines in *fmr1-/y* mice and a decrease in small spines compared to wild-type. Chi-squared test, *p* < 0.0001. Although the difference in the mean area of wild-type and *fmr1-/y* spines did not reach statistical significance **(E)**; 3 WT v 3 *fmr1-/y* mice, unpaired *t*-test, *p* = 0.157), binning the data from all spines (221 WT v 224 *fmr1-/y*), revealed a rightward shift in the data distribution, due to a higher incidence of larger spines in *fmr1-/y* compared to wild-type **(F)**. Chi-squared test, *p* = 0.0002.

In addition to the higher spine number, we uncovered a significant increase in the total length of *fmr1-/y* spines (Figures 6A–C) in the accumbens (wild-type = 856 ± 4 nm, *fmr1-/y* = 1069 ± 20 nm; *p* = 0.001). This manifest as a greater number of spines longer than 1 μm (wild-type = 60/221, *fmr1-/y* = 115/224) and fewer spines shorter than 1 μm (wild-type = 161/221, *fmr1-/y* = 109/224) in *fmr1-/y* accumbens compared to wild-type (Figure 6D). Interestingly, the differences in total cross-sectional spine area did not reach statistical significance (*p* = 0.157) between wild-type (0.22 ± 0.02 μm^2^) and *fmr1-/y* (0.27 ± 0.02 μm^2^) (Figure 6E). However, when total spine area was pooled within genotypes and the Data distribution analyzed, there was a significant increase in the number of larger spines observed in *fmr1-/y* mice (Chi^2^ test *p* = 0.0002; Figure 6F).

**Figure 7:**
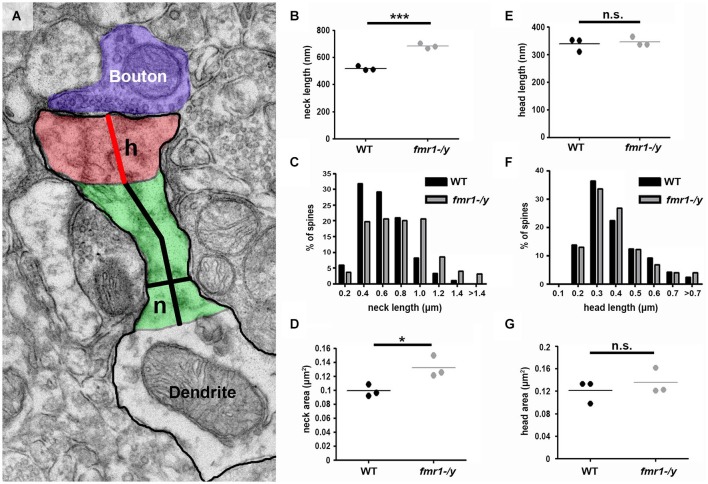
**Increased spine neck area and length in *fmr1-/y* accumbens. (A)** Representative electron micrograph annotated with measurement parameters, including neck length (long black line), neck width (short black line), head length (red line), neck area (green) and head area (red). **(B)** Mean neck length of wild-type spines (221 spines, *n* = 3 mice) is significantly longer than *fmr1-/y* mice (224 spines, *n* = 3 mice). Unpaired *t*-test, *p* = 0.0003. **(C)** Binning all the spines from each genotype reveals a higher incidence of longer necks in the *fmr1-/y* mice compared to wild-type. Chi-squared test, *p* < 0.0001. **(D)** The area of *fmr1-/y* spine necks was significantly larger than those of wild-type spines. Unpaired *t*-test, *p* = 0.031. In striking contrast to the changes in *fmr1-/y* spine necks, the spine heads were not different in length **(E)**; unpaired *t*-test *p* = 0.684. **(F)**; chi-squared test *p* = 0.795) or in area **(G)**; unpaired *t*-test *p* = 0.48).

**Figure 8:**
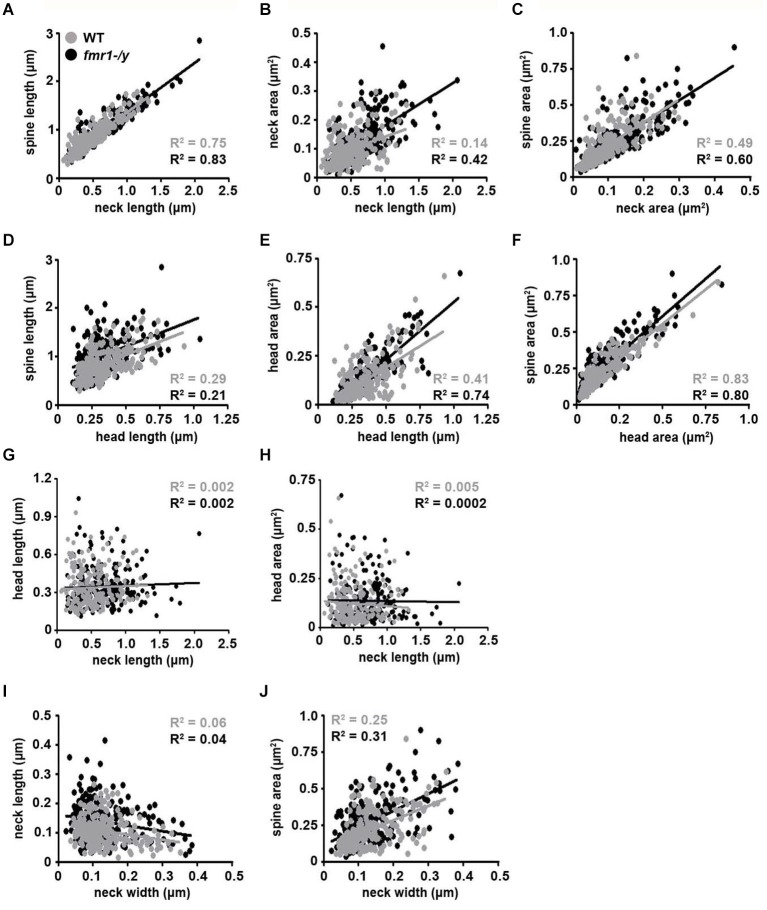
**Correlation analysis of morphological parameters of dendritic spines**. Representative dot plots of spine morphological data. Data points representing individual spines from wild-type mice are highlighted with black dots and best-fit trend lines are black. Data points from *fmr1-/y* mice are highlighted in gray dots and best-fit trend lines are gray (*n* = 221 wild-type spines, *n* = 224 *fmr1-/y spines)*. All Pearson *R*^2^ values are color-coded as above.

The observed spine elongation on average by 25% could be due to alterations in neck length, head length or both (Figure 7A). An analysis of more than 220 intact spines per genotype revealed significantly longer spine necks in the *fmr1-/y* mice (684 ± 11 nm) compared to wild-type (518 ± 10 nm; *p* = 0.0003), due to a significantly greater number of long spines in the *fmr1-y* mice (Chi^2^ test *p* < 0.0001; Figures 7B,C). In contrast, spine neck width was similar between genotypes (wild-type = 140 ± 4.3 nm, *fmr1-/y* = 130 ± 9.8 nm, *p* = 0.79). Neck length correlated with spine length and neck area (Figures 8A,B). Neck area correlated with spine area (Figure 8C) and, accordingly, a larger spine neck area was found in *fmr1-/y* mice (0.13 ± 0.009 μm^2^) compared to wild-type (0.099 ± 0.005 μm^2^; *p* = 0.031) (Figure 7D). Conversely, no change in spine head length (wild-type = 338 ± 14 nm, *fmr1-/y* = 346 ± 10 nm; *p* = 0.68) or head area (wild-type = 0.12 ± 0.012 μm^2^, *fmr1-/y* = 0.14 ± 0.013 μm^2^; *p* = 0.48) was observed (Figures 7E–G). To investigate whether the specific alteration in spine neck morphology modified the relationship of distinct morphological parameters in the mouse model of Fragile X syndrome, we performed a detailed correlation analysis. In agreement with the fact that spine heads generally constitute the major bulk of spines (Arellano et al., 2007), spine head area correlated more strongly with total spine area than spine necks (Figures 8C,F). Nevertheless, there was still positive correlation between neck area and the total spine area (Figure 8C). Weak positive correlation was observed between neck width and spine area (Figure 8J). No correlation was found between neck length and head length (Figure 8G), neck length and head area (Figure 8H), and only a very weak negative correlation was found between neck width and neck length (Figure 8I). These data are remarkably similar to data recently reported in spines of living neurons imaged in wild-type mouse hippocampus (Tønnesen et al., 2014). Importantly, while both genotypes showed similar correlation between neck length and neck area (Figure 8B), and head length and head area (Figure 8E), the strength of correlation was significantly stronger in the *fmr1-/y* spines. Together with the distinct level of correlation between the head and neck lengths and the total spine length, these analyses point to the weighted contribution of the spine neck in determining total spine length (Figures 8A,D respectively). Collectively, these data (summarized in Table 1) reveal that FMRP loss leads to an increase in spine density and a specific elongation of the spine neck, but not the spine head in the accumbens.

**Table 1:** **Spine analysis parameters and results**.

	Genotype	Range	Mean	Median	Interquartile Range	*n (spines/animals)*
**PSD density**	WT	0.066–0.53	0.25	0.23	0.2–0.3	150 images/3
*p* = 0.049	*fmr1-/y*	0.066–0.72	0.32	0.3	0.23–0.39	150 images/3
**PSD length**	WT	106–639	275	262	210–318	300/3
*p* = 0.42	*fmr1-/y*	124–659	289	273	216–346	300/3
**Spine length**	WT	292–1783	856	801	664–1057	221/3
*p* = 0.001	*fmr1-/y*	339–2838	1030	1012	745–1255	224/3
**Spine area**	WT	0.05–0.84	0.21	0.18	0.12–0.29	221/3
*p* = 0.157	*fmr1-/y*	0.03–0.82	0.28	0.25	0.17–0.35	224/3
**Neck length**	WT	68–1319	518	484	334–646	221/3
*p* = 0.0003	*fmr1-/y*	122–2072	685	656	411–873	224/3
**Neck area**	WT	0.01–0.3	0.099	0.087	0.05–0.13	221/3
*p* = 0.031	*fmr1-/y*	0.009–0.45	0.13	0.11	0.08–0.17	224/3
**Neck width**	WT	35–351	136	121	89–166	221/3
*p* = 0.79	*fmr1-/y*	23–384	133	113	82–162	224/3
**Head length**	WT	122–932	338	299	229–421	221/3
*p* = 0.68	*fmr1-/y*	112–1046	346	243	243–413	224/3
**Head area**	WT	0.017–0.66	0.12	0.089	0.05–0.17	221/3
*p* = 0.48	*fmr1-/y*	0.011–0.67	0.14	0.1	0.06–0.18	224/3

*Length in nm, area in um^2^ and density in PSD/um^2^*.

Changes to the postsynapse may be mirrored by alterations in the presynapse, which can affect synaptic transmission and may explain some of the effects observed in our physiology experiments. To this end, we measured both the presynaptic active zone length and the total number of vesicles in 50 boutons per animal. These experiments revealed a similar correlation between active zone length and vesicle number in both strains (wild-type *R*^2^ = 0.49, *fmr1-/y R*^2^ = 0.39), but showed no difference in the presynaptic parameters measured. Active zone length was similar between strains (wild-type = 183.9 ± 8.9 nm, *fmr1-/y* = 195.1 ± 0.5 nm; *p* = 0.28) and the total boutonal vesicle number showed no difference (wild-type = 30 ± 0.3, *fmr1-/y* = 33 ± 1.8; *p* = 0.21).

Given the importance of spine morphology on synaptic physiology (Nimchinsky et al., 2002; Sala and Segal, 2014; Tønnesen et al., 2014), the increased density and elongation of spines in the *fmr1-/y* accumbens is likely to contribute to the disrupted synaptic plasticity in the mouse model of Fragile X syndrome.

## Discussion

In this study, we combined electrophysiological and electron microscopy methods and searched for functional and structural synaptic deficits in the nucleus accumbens of *fmr1-/y* mice. Our main findings are that 1/ LTP is ablated at excitatory accumbens synapses of *fmr1-/y* mice while there is a parallel increase of the AMPA/NMDA ratio and 2/ that adult *fmr1-/y* mice have significantly more excitatory synapses in the accumbens core and that spine necks were significantly longer in these mice.

Glutamatergic accumbens synapses express a wide array of mechanistically diverse forms of LTDs: NMDAR-dependent LTD, mGluR5-dependent and endocannabinoid-mediated retrograde LTD and presynaptic mGluR2/3 auto receptor-mediated LTD (Robbe et al., 2002b,c). In support of the idea that synaptic plasticity deficits occur in the accumbens of *fmr1-/y* mice, we recently reported that the endocannabinoid/mGluR5 signaling complex and associated long-term depression are profoundly perturbed at accumbens synapses of *fmr1-/y* (Jung et al., 2012). Here we first established a STDP paradigm capable to induce a strong and reliable LTP of synaptic efficacy at excitatory accumbens synapses. Because a reliable STDP LTP-inducing protocol at accumbens synapses was lacking (Ji and Martin, 2012), our study significantly expands previous work (Pennartz et al., 1993; Kombian and Malenka, 1994; Robbe et al., 2002b; Schotanus and Chergui, 2008b; Ji and Martin, 2012).

Accumbal STDP LTP was induced when the presynaptic activity precedes the spiking of the postsynaptic cell, following a hebbian rule (Caporale and Dan, 2008). In the present study GABA-A mediated transmission was blocked to isolate the glutamatergic synapses (see also Pawlak and Kerr, 2008). GABAergic IPSPs arising from fast-spiking, low threshold-spiking interneurons and collaterals can modify the back-propagating action potentials and the timing rules of STDP (Fino and Venance, 2010). Thus, future studies will be needed to test the importance of GABAergic activity on this LTP in pathophysiological conditions.

In wild-type mice, spike timing-dependent LTP was prevented by bath-application of D-AP5, showing its dependency on NMDAR. This result is in accordance with the majority of studies on STDP, where NMDAR-mediated LTP is found when the presynaptic activity precedes the spiking of the postsynaptic cell following a hebbian rule (Caporale and Dan, 2008 ; but see Paille et al., 2013).

Importantly, we found that LTP was ablated in the accumbens of *fmr1-/y* mice. This result is in line with reports of a lack of LTP in other brain structures of *fmr1-/y* mice (Meredith et al., 2007; Wilson and Cox, 2007; Suvrathan and Chattarji, 2011). A single previous study showed a deficient LTP in the PFC of *fmr1-/y* mice, which was attributed to a down regulation in dendritic L-type VGCC and unreliable dendritic Ca^2+^ signaling (Meredith et al., 2007). Noteworthy, L-type channels are not needed for the expression of LTP in neither dorsal nor ventral striatum (Ji and Martin, 2012; Paille et al., 2013), but rather necessary for the expression of timing-dependent long-term depression. Thus, a deregulation of L-type VGCC expression is not expected to have a negative effect on LTP.

A differential synaptic expression of NMDAR can set the threshold and capacity to express NMDAR-dependent LTP. This idea is supported by several studies in *fmr1-/y* mice showing that defects in LTP coincide with decreased NMDAR protein levels or changes in the AMPA/NMDA ratio (Harlow et al., 2010; Yun and Trommer, 2011). Interestingly, FMRP binds GluN1, GluN2A, and GluN2B mRNAs and that the loss of FMRP leads to deregulated translation of these NMDAR subunits (Schütt et al., 2009; Edbauer et al., 2010) as well as abnormal expression of NMDAR (Krueger et al., 2011). Since a decreased NMDA expression can go along with deficits in LTP (Harlow et al., 2010; Bostrom et al., 2015), we tested whether the defects in LTP are accompanied by changes in AMPA/NMDA ratio. In agreement with several other studies (Harlow et al., 2010; Yun and Trommer, 2011), we found an increase of the AMPA/NMDA ratio in *fmr1-/y* mice. Since the basal spontaneous AMPAR transmission and the size of the PSD length were unchanged (see Figures 8C,D and discussion below) this increase might indicate a reduction in NMDAR transmission. Our observation of reduced amplitude of basal spontaneous NMDAR synaptic currents supports this interpretation (Figure 4E). Alterations in the synaptic signaling machinery leading to the lack of LTP could be linked to structural abnormalities. Although the direct influence of spine morphology on LTP has not widely been studied, aberrant dendritic arborization and abnormal spine structures are associated with synaptic plasticity deficits in Fragile X as in other types of mental retardation (Penzes et al., 2011). We conducted an ultrastructural analysis by electron microscopy and discovered a number of spine alterations in adult *fmr1-/y* mice. Firstly, we found that adult *fmr1-/y* mice have significantly more excitatory synapses in the accumbens core. A recent observation based on light microscopic analysis revealed that the trend towards an increased spine density in *fmr1-/y* mice treated with cocaine, but not in vehicle did not reach statistical significance (Smith et al., 2014). In the present study, we used electron microscopy to unequivocally identify those dendritic spines, which receive asymmetrical synapses and the 3D analysis uncovered a significantly increased incidence of unusually long spines in *fmr1-/y* mice. These findings of extra spines and elongated spines in the *fmr1-/y* mice, are in agreement with the long filopodial spines observed in other brain areas of *fmr1-/y* mice (Meredith et al., 2007; Cruz-Martín et al., 2010; He and Portera-Cailliau, 2013) and in Fragile X post-mortem tissue (Irwin et al., 2001), and can be interpreted as spine immaturity, due to a deficit in synapse pruning (Bagni and Greenough, 2005). In fact, many of our spine measurements are similar to those reported in various studies from distinct brain regions. For example, we found that spine neck width was the same between genotypes, which is consistent with findings in the cerebellum of *fmr1-/y* mice (Koekkoek et al., 2005). Furthermore, in both genotypes, neck diameter showed a weak, but significant correlation with total spine volume, which was previously shown in the mouse neocortex (Arellano et al., 2007).

Given our discovery of ablated LTP in the *fmr1-/y* mice, it is interesting to note that spine necks were significantly longer in these mice. It may therefore be reasonable to speculate that the increased incidence of long immature-appearing spines could be due to a lack of spine pruning, combined with a loss of LTP-induced spine shrinkage. It is also important to note that the spine alteration was specifically observed within the neck and not the head compartment. Previous work in the mouse neocortex has shown that the spine neck length negatively correlated with the strength of somatically recorded membrane potential changes following glutamate uncaging next to the spine head (Araya et al., 2006). The study showed that the effect was independent of spine proximity to the soma or spine head size, as both long and short spines had similar head sizes. This shows that specific changes in spine neck length are sufficient to filter synaptic activity. Therefore, a higher incidence of long necked spines in the *fmr1-/y* mice may lead to increased filtering of synaptic inputs resulting in a lack of LTP.

So far it cannot be determined, whether the structural differences are a cause or a consequence of the lack of LTP in *fmr1-/y*. Recent studies in the neocortex reported a faster turnover of dendritic spines in *fmr1-/y* mice (Cruz-Martín et al., 2010; Padmashri et al., 2013). This could be interpreted as a failure of spine stabilization due to the lack of LTP. Our electron microscopy data could be a snapshot of a similar alternation in the accumbens. In addition, the lack of LTD in the nucleus accumbens (Jung et al., 2012), which has been shown to be necessary for the pruning of neurons in other brain structures (Bastrikova et al., 2008) could prevent the elimination of spines and lead to the overabundance of long and immature spines in *fmr1-/y* mice. In return, the changes in the density and geometry of postsynaptic spines most probably have an influence on the chemical and electrical compartmentalization and thereby on action potential back-propagation and synaptic integration (Tønnesen et al., 2014; Wijetunge et al., 2014).

Fragile X patients show behavioral symptoms such as attention deficit and hyperactivity, social anxiety and an overall stressful disposition and importantly, *fmr1-/y* mice show corresponding behaviors (Kooy, 2003; Tranfaglia, 2011). Together, our study reveals new structural and functional alterations in the nucleus accumbens of *fmr1-/y* mice and suggests potential synaptic substrates of social interaction deficits and emotional problems observed in Fragile X.

## Conflict of Interest Statement

The authors declare that the research was conducted in the absence of any commercial or financial relationships that could be construed as a potential conflict of interest.
